# Association study with Wegener granulomatosis of the human *phospholipase Cγ2 *gene

**DOI:** 10.1186/1477-5751-4-1

**Published:** 2005-02-09

**Authors:** Peter Jagiello, Stefan Wieczorek, Philipp Yu, Elena Csernok, Wolfgang L Gross, Joerg T Epplen

**Affiliations:** 1 Department of Human Genetics, Ruhr-University Bochum Germany; 2 Institute of Medical Microbiology, Immunology and Hygiene, Technical University Munich, Germany; 3 Department of Rheumatology, University Hospital Luebeck and Rheumaklinik Bad Bramstedt, Germany

## Abstract

**Background:**

Wegener Granulomatosis (WG) is a multifactorial disease of yet unknown aetiology characterized by granulomata of the respiratory tract and systemic necrotizing vasculitis. Analyses of candidate genes revealed several associations, *e.g. *with *α*(1)-antitrypsin, proteinase 3 and with the *HLA-DPB1 *locus. A mutation in the abnormal limb mutant 5 (ALI5) mouse in the region coding for the hydrophobic ridge loop 3 (HRL3) of the *phospholipaseCγ2 *(*PLCγ-2*) gene, corresponding to human *PLCγ*-2 exon 27, leads to acute and chronic inflammation and granulomatosis. For that reason, we screened exons 11, 12 and 13 coding for the hydrophobic ridge loop 1 and 2 (HRL1 and 2, respectively) and exon 27 of the PLCγ-2 protein by single strand conformation polymorphism (SSCP), sequencing and PCR/ restriction fragment length polymorphism (RFLP) analyses. In addition, we screened indirectly for disease association via 4 microsatellites with pooled DNA in the *PLCγ*-2 gene.

**Results:**

Although a few polymorphisms in these distinct exons were observed, significant differences in allele frequencies were not identified between WG patients and respective controls. In addition, the microsatellite analyses did not reveal a significant difference between our patient and control cohort.

**Conclusion:**

This report does not reveal any hints for an involvement of the *PLCγ*-2 gene in the pathogenesis of WG in our case-control study.

## Background

Wegener granulomatosis (WG) is a systemic inflammatory disease of unknown aetiology characterized by granulomata of the respiratory tract and systemic necrotizing vasculitis [1]. There is a strong and specific association with presence of anti-neutrophil cytoplasmatic antibodies to a defined target antigen, proteinase 3 (PR3-ANCA), which is present within primary azurophil granules of neutrophils (PMN) and lysozymes of monocytes [2]. Upon cytokine priming of PMNs, this enzyme translocates to the cell surface, where PR3-ANCAs can interact with their antigens and activate PMNs [3]. It has been shown that PMNs from patients with active WG expressing PR3 on their surfaces produce respiratory burst and release proteolytic enzymes after activation with PR3-ANCA [4]. The consequence is a self-sustaining chronifying inflammatory process. WG appears as a multifactorial disease, and environmental influences still remain elusive. Several factors, such as bacterial infections, have been proposed as probable initiators of the disease [5]. It has been reported that chronic carrier status of *Staphylococcus aureus *is a risk factor for disease exacerbation in WG [6]. Recently a strong association of WG with distinct *HLA-DPB1 *alleles or rather an extended haplotype, respectively, in the MHC class II region has been reported [7]. In addition, analyses of candidate genes revealed several associations, *e.g. *with *α*(1)-antitrypsin, and proteinase 3 [[8] and [9]].

A primary candidate gene, *PLCγ*-2, was proposed on the basis of a novel animal model system. A mutation in the abnormal limb mutant 5 (ALI5) mouse [10] causes a phenotype comparable to human autoimmunity disease like WG: inflammations, granulomatosis, affected organs (lung, kidney with glomerulonephritis, eye and skin) and ANCAs were detected in the ALI5 mouse initially. Yet, this result has to be confirmed in further studies. The ALI5 mutation is located in the genomic region coding for the hydrophobic ridge loop 3. In mouse *PLCγ-2 *mutation leads to acute and chronic inflammations with a phenotype comparable to WG. Therefore, human *PLCγ-2 *is a good candidate gene for seeking predisposing genetic factors for WG. The human gene is a member of the PLC family comprising 12 closely related molecules involved in signal transduction from numerous receptors [11]. The protein is activated by cytoplasmatic tyrosine kinases (Lyn, Syk, and Btk) which are induced by engagement of B-cell receptors. In turn, the PLCγ-2 activation leads to the generation of diacylglycerol (DAG) and inositol 1,4,5-trisphosphat (IP3). While DAG activates protein kinase C (PKC, [12]), IP3 mediates Ca^2+ ^mobilization, which is required for activation of B-cells [13]. PLCγ-2 itself is expressed mainly in B-cell, hematopoetic cells, macrophages, granulocytes, testis, sperm, skin and brain [14]. The protein consists of two catalytic domains, which are separated by two SH2 and one SH3 domains [15]. It was established that PLCγ-2 mediates the coupling of G-protein-coupled receptors (GPCRs) and Ca^2+ ^entry in cell lines [16]. The human *PLCγ-2 *gene is localized on chromosome 16 (16q24.1) spanning 179 kb of genomic DNA. The 4.2 kb mRNA consists of 33 Exon coding for a M_*r *_140,000 protein with 1265 amino acids [17]. *PLCγ-2 *is highly polymorphic [18].

Here, we report on an indirect association screen by microsatellite analysis and pooling of DNA in a case-control study design. Furthermore, we screened exons 11, 12 and 13, partly coding for the hydrophobic ridge loop 1 and 2 of PLCγ-2 protein, by single stranded conformation polymorphism (SSCP), sequencing and the PCR/RFLP method. As the ALI5 mutation is located in the region corresponding to the human exon 27, this exon was also screened by SSCP.

## Results

### Analyses of microsatellites

**Figure 1:**
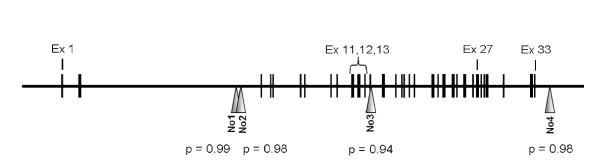
Schematic representation of the human PLCγ-2 gene with relative localization of exons and investigated microsatellites. P values were generated by contingency tables (for details see "materials and methods"). Vertical lines: exons; No1-6: investigated microsatellites 1-6; Ex: exon.

In the present study we analysed 4 microsatellites intra- or juxta-genic of the *PLCγ-2 *gene with pooled DNAs from WG patients compared with those from controls (table 1; figure 1). All markers exhibited at least 3 alleles and did not show any intra-subgroup differences. The microsatellite analyses did not reveal significantly different allele distributions between WG patient and control pools (figure 1).

**Table 1:** Primer sequences and information about microsatellites used in study

	Primer sequence		
		
No.	sense^1^	antisense	*nucleotide marker
1	CGCACATGTATCCAGAACT	AGAGGTGGACCCATGCTTA	*di (AT)
2	CAAAGAAGATAAGGGCAGGC	CCTAGGCGACTCAGTGAGACT	*tetra (TTTA)
3	AGGAGTTCGAGAAGAGCCTG	TGCCACTACACCCAGATGAT	*di (AC)
4	TGATCTGTGTCTGGGCTTTC	AGTTGTGACCCTAACATTGCA	*di (AC)

^1 ^The fluorescence labelled tail (5-Fam-CATCGCTGATTCGCACAT) was added to the 5'end of each sense primer

### SSCP, sequencing and PCR/RFLP analyses

SSCP analysis was chosen to identify mutations or polymorphisms, respectively, in exons 11, 12, 13 and 27 of the *PLCγ-2 *gene. DNA's of patients and controls with altered migration behaviour were sequenced. Identified variations were genotyped individually by SSCP and/or PCR/RFLP in individual patient and control samples (see table 2). Exon 12 revealed a low frequent SNP (1122G>A), which represents the first base pair of the exon. In exon 13 two previously identified SNPs (1293T>C and 1296T>C) were detected [18]. Both SNPs showed similar frequencies as reported [18]. All variations were found not significantly different in WG patients compared to the control group, and they were not associated with amino acid substitutions. In one patient our analyses revealed a single base substitution (3030G>A) in exon 27.

**Table 2:** Summary of found variations in the human *PLCγ-2 *gene in exons 11, 12, 13 and 27

	variation		frequency of alleles			
				
exon	bp^1^	codon	WG patients	controls	P value	restriction enzymes
11	-	-	-	-	-	-
12	1122G>A	T329T	3/262	3/162	0.55	BanI
13	1293T>C^2^	F382F	7/350	4/324	0.68	PfIFI
	1296T>C^2^	D383D	143/262	96/186	0.78	TaqI
27	3030G>A	T961T	1/166^3^	0/180^3^	0.51	-

^1^Numbering according to BC007565 (UCSC); ^2^Previously reported SNPs; ^3^Frequencies determined by SSCP analyses

## Discussion

As recently reported a mutation in the *PLCγ*-2 gene in the ALI5 mouse leads to acute and chronic inflammation and granulomatosis. In addition, the ALI5 mice show similar symptoms as WG patients. For this reason screening of *PLCγ*-2 appears as a logical consequence in seeking candidate genes for WG. In the present study *PLCγ*-2 was analysed by an indirect microsatellite approach using pooled DNA from WG patients with a defined PR3-ANCA^+ ^status and a matched control cohort as reported before [7]. Furthermore, exons partly representing the catalytic domains of the PLCγ-2 protein, HRL1, 2 and 3, were analysed by SSCP, sequencing and by the PCR/RFLP method. We did not find any hints of an involvement of the *PLCγ*-2 gene in the pathophysiology of WG. Hence, we exclude at this time that the *PLCγ*-2 gene predisposes for WG in our cohort.

Our study revealed 4 single base substitutions, 2 of which were reported before [18]. A further silent and low frequent SNP was detected in exon 12 which did not differ significantly between patient and control cohorts after PCR/RFLP analyses. As this SNP is the first basepair (bp) after the 3'-splice site one might hypothesise an influence on splicing but to present knowledge this SNP does not change a consensus sequence required for splicing. The ALI5 mutation is located in the HRL3 domain partly corresponding to the human exon 27 of *PLCγ*-2. Our analysis revealed a SNP (G>A) at position 3030 in one WG patient.

The indirect microsatellite based approach did not reveal any association of *PLCγ*-2 with WG. Altogether 4 microsatellites spread in the *PLCγ*-2 gene were analysed. The approach using pooled DNA and *ad hoc *designed markers intragenic or in the immediate vicinity of a distinct gene has proven to be a reliable and efficient method to detected predisposing loci in WG before [7]. Here, none of the markers did show a significant allele distribution between the patient and control group.

## Conclusion

In conclusion, our analysis of the human *PLCγ*-2 gene did not reveal an association of *PLCγ*-2 with WG. In contrast to ALI5 mice, where a single mutation leads to distinct symptoms of inflammatory autoimmunity, human WG depends on a more complex genetic background. Further analysis of all exons of *PLCγ*-2 might yield an association with WG but our microsatellite analysis strongly suggests that predisposition for WG is not due to variations in the *PLCγ*-2 gene.

## Material and Methods

### Patients and controls

175 well-characterized patients of German origin with a clinical diagnosis of WG and a defined PR3-ANCA^+ ^status were included in present study. Diagnosis of WG was established according international standards [[19] and [20]]. All patients were biopsy-proven. Biopsies were seen in German reference centre for vasculitis (Department of Pathology, University of Schleswig-Holstein Campus Luebeck, Germany) by 2 different observers. A group of 165 healthy individuals of German origin were used as controls. All persons gave informed consent.

### Microsatellite analysis

Pooling of DNA was performed as reported before [7]. Patient (n = 150) and healthy control (n = 100) individuals from the abovementioned groups were divided into 3 and 2 sub-pools, respectively, containing 50 persons each.

In this study 3 intragenic microsatellites as well as one in the immediate vicinity of the gene were included (table 1; see also UCSC Database, June 2002 Freeze; URL). Oligonucleotides were designed by Primer Express 2.0 software (ABI) adjusted to an annealing temperature of 55°C.

For PCR we used the 'tailed primer PCR' as described before [7]. For amplification three oligonucleotides were used: 1. tailed sense primer (tailed F), 2. anti-sense primer and 3. labeled primer (labeled F) corresponding to the 5'-tail sequence of tailed F. PCR conditions were as follows: 1 × PCR buffer (Qiagen), 1.5 pmol labeled F, 0.2 mM each dNTP, 3 mM MgCl_2_, 0.2 pmol tailed F, 1.5 pmol reverse primer, 0.25 U Qiagen Hot Start Taq (Qiagen) and 50 ng DNA.

Electrophoreses were run using ABI standard protocols. Raw data were analyzed by the Genotyper software (ABI) producing a marker-specific allele image profile (AIP, see [21] and [7]). AIP consists of series of peaks with different heights reflecting the allele frequency within each analyzed DNA pool.

Statistics for comparisons of allele frequencies of patients and controls was performed as described before [[21] and [7]]. Case and control distributions were compared statistically by means of contingency tables.

### SSCP, sequencing and PCR/RFLP analyses

Exons 11, 12, 13 and 27 were analysed by the SSCP method. PCR was performed using oligonucleotides reported before ([18], exon 27 corresponding to exon 26). Heat-denaturated fragments were then separated by polyacrylamide gel electrophoresis under non-denaturating conditions yielding specific band patterns for each of the alleles. Results were visualized by autoradiography. Alleles of representative probes were determined by direct DNA sequence analysis on a 377 ABI automatic sequencer (ABI). Afterwards, variations were genotyped individually by PCR/RFLP method with restriction enzymes specific for the respective change (for details see table 2). The variation in exon 27 was individually genotyped by the SSCP method.

## Authors' contribution

PJ and SW carried out the molecular genetic studies, performed the statistical analysis and drafted the manuscript. PY participated in study design and helped to draft the manuscript. EC and WLG provided the samples and performed diagnostics of the patient group. JTE conceived of the study, and participated in its design and coordination and helped to draft the manuscript. All authors read and approved the final manuscript.
